# Supplementary material for: Comparative Analysis of Antioxidant Accumulation under Cold Acclimation, Deacclimation and Reacclimation in Winter Wheat
Source: Plants (Basel). 2022 Oct 23;11(21):2818. doi: 10.3390/plants11212818 (PMC9659242; doi:10.3390/plants11212818)
Supplement: Supplementary file 1 [file plants-11-02818-s001.zip › Supplementary_material.pdf]

plants

MDPI

Supplementary material

Comparative analysis of antioxidant accumulation under cold acclimation, deacclimation and reacclimation in winter wheat

Table S1. Descriptive statistics and Wilcoxon rank sum test results of antioxidant concentration values between tissues in constant low-temperature (CLT) and prolonged higher low-temperature (PHLT) treatments.

		CTRL			CA			DEA			REA		
**Treatment**	**Antioxidant**	**Leaf mean ± SD**	**Crown mean ± SD**	**p-value**	**Leaf mean ± SD**	**Crown mean ± SD**	**p-value**	**Leaf mean ± SD**	**Crown mean ± SD**	**p-value**	**Leaf mean ± SD**	**Crown mean ± SD**	**p-value**
CLT	Total ascorbate (nmol/mg FW)	4.48 ± 0.76	1.62 ± 0.81	8.65E-07 ***	14.01 ± 1.30	4.74 ± 1.37	4.81E-07 ***	8.01 ± 1.03	2.53 ± 0.55	3.22E-07 ***	8.21 ± 0.75	2.64 ± 0.37	3.22E-07 ***
	ASC (nmol/mg FW)	3.01 ± 0.46	2.39 ± 0.79	0.009912 **	10.67 ± 1.38	5.15 ± 0.89	4.41E-10 ***	6.55 ± 1.21	2.72 ± 0.51	3.11E-07 ***	7.93 ± 1.18	2.44 ± 0.367	3.22E-07 ***
	DHA (nmol/mg FW)	1.47 ± 0.44	NA ± NA	NA	3.34 ± 0.85	0.19 ± 0.09	0.007717 **	1.46 ± 0.63	0.18 ± 0.14	0.02735 *	0.76 ± 0.37	0.23 ± 0.17	1.94E-04 ***
	ASC / DHA ratio	2.26 ± 0.84	NA ± NA	NA	3.52 ± 1.38	34.31 ± 21.92	0.001504 **	5.90 ± 4.05	23.15 ± 19.52	0.04211 *	12.28 ± 8.06	30.84 ± 54.86	0.4221
	Total glutathione (pmol/mg FW)	218.94 ± 51.20	327.29 ± 152.05	0.01418 *	1071.53 ± 232.70	738.68 ± 157.79	2.19E-05 ***	771.49 ± 166.18	642.88 ± 187.51	0.05086	1052.99 ± 322.03	1221.19 ± 270.75	0.07359
	GSH (pmol/mg FW)	173.06 ± 51.73	279.83 ± 151.50	0.0224 *	951.45 ± 226.35	675.23 ± 163.52	0.001106 **	711.41 ± 155.45	619.97 ± 186.62	0.1401	903.60 ± 257.63	1017.54 ± 231.55	0.1427
	GSSG (pmol/mg FW)	22.94 ± 16.32	29.37 ± 14.59	0.05939	60.04 ± 33.81	33.15 ± 14.94	0.01585 *	29.59 ± 19.34	9.07 ± 7.03	7.44E-05 ***	74.70 ± 46.20	101.82 ± 50.98	0.1341
	GSH / GSSG ratio	17.28 ± 33.38	13.25 ± 13.77	0.6702	22.12 ± 14.37	26.38 ± 16.43	0.5115	57.62 ± 114.87	392.34 ± 1121.34	0.0001278 ***	16.46 ± 10.63	13.98 ± 12.34	0.1427
	NAD^+ (pmol/mg FW)	24.64 ± 15.66	60.86 ± 19.75	0.0001741 ***	42.68 ± 26.54	109.22 ± 16.54	2.06E-08 ***	24.64 ± 13.62	62.77 ± 23.82	1.55E-05 ***	39.12 ± 14.70	96.91 ± 19.84	8.82E-10 ***
	$NADP^+$ (pmol/mg FW)	6.05 ± 3.27	7.83 ± 5.04	0.331	15.70 ± 9.19	9.87 ± 3.70	0.04103 *	1.69 ± 1.83	4.17 ± 2.18	0.006712 **	3.04 ± 2.09	7.44 ± 2.13	4.10E-05 ***
PHLT	Total ascorbate (nmol/mg FW)	4.48 ± 0.76	1.62 ± 0.81	8.65E-07	7.29 ± 0.63	2.03 ± 0.36	3.21E-07 ***	7.17 ± 0.78	1.51 ± 0.38	3.22E-07 ***	7.22 ± 0.66	1.50 ± 0.42	3.22E-07 ***
	ASC (nmol/mg FW)	3.01 ± 0.46	2.39 ± 0.79	0.009912 **	6.47 ± 0.94	2.17 ± 0.37	2.20E-10 ***	6.16 ± 0.93	1.66 ± 0.40	3.22E-07 ***	7.61 ± 0.92	1.42 ± 0.38	2.20E-10 ***
	DHA (nmol/mg FW)	1.47 ± 0.44	NA ± NA	NA	0.96 ± 0.64	0.10 ± 0.03	0.004954 **	1.07 ± 0.60	0.04 ± 0.01	0.0117 *	0.46 ± 0.27	0.12 ± 0.07	0.0491 *
	ASC / DHA ratio	2.26 ± 0.84	NA ± NA	NA	26.85 ± 69.17	20.78 ± 7.58	0.02188 *	11.62 ± 19.31	40.91 ± 26.54	0.07018	32.93 ± 41.68	19.12 ± 17.94	0.457
	Total glutathione (pmol/mg FW)	218.94 ± 51.20	327.29 ± 152.05	0.01418 *	683.48 ± 168.46	603.48 ± 164.18	0.2292	563.82 ± 59.98	538.17 ± 107.46	0.4235	800.23 ± 211.32	678.67 ± 213.08	0.09706
	GSH (pmol/mg FW)	173.06 ± 51.73	279.83 ± 151.51	0.0224 *	652.07 ± 172.27	547.14 ± 149.94	0.1437	540.07 ± 66.92	535.11 ± 106.30	0.9145	739.99 ± 183.74	584.22 ± 195.21	0.01028 *
	GSSG (pmol/mg FW)	22.94 ± 16.32301	29.37 ± 14.59	0.05939	20.24 ± 19.47	8.02 ± 5.71	0.0196 *	14.87 ± 12.04	6.22 ± 3.82	0.03764 *	22.81 ± 15.41	47.23 ± 22.73	0.0004675 ***
	GSH / GSSG ratio	17.28 ± 33.38	13.25 ± 13.77	0.6702	66.11 ± 64.79	146.88 ± 199.42	0.1006	115.25 ± 179.23	134.50 ± 110.42	0.05677	47.33 ± 30.95	15.12 ± 8.30	1.54E-06 ***
	NAD^+ (pmol/mg FW)	24.64 ± 15.66	60.86 ± 19.75	0.0001741 ***	35.82 ± 14.78	58.49 ± 19.48	0.0008653 ***	20.01 ± 14.94	40.69 ± 14.05	0.0005828 ***	39.90 ± 17.90	56.05 ± 13.61	0.009631 **
	$NADP^+$ (pmol/mg FW)	6.05 ± 3.27	7.83 ± 5.04	0.331	13.06 ± 6.26	2.024 ± 1.40	7.32E-07 ***	10.64 ± 6.21	2.65 ± 1.86	0.0001018 ***	9.11 ± 4.75	2.96 ± 2.04	3.69E-05 ***

** indicates significant differences at p < 0.05, ** at p < 0.01, and *** at p < 0.001.*

Table S2. Descriptive statistics and Wilcoxon rank sum test results of antioxidant concentration values between constant low-temperature (CLT) and prolonged higher low-temperature (PHLT) treatments in leaf and crown tissues.

		CA			DEA			REA		
**Tissue**	**Antioxidant**	**CLT mean ± SD**	**PHLT mean ± SD**	**p-value**	**CLT mean ± SD**	**PHLT mean ± SD**	**p-value**	**CLT mean ± SD**	**PHLT mean ± SD**	**p-value**
LEAF	Total ascorbate (nmol/mg FW)	14.01 ± 1.30	7.29 ± 0.63	3.22E-07 ***	8.01 ± 1.03	7.17 ± 0.78	0.01137 *	8.21 ± 0.75	7.22 ± 0.66	1.81E-04 ***
	ASC (nmol/mg FW)	10.67 ± 1.38	6.47 ± 0.94	2.20E-10 ***	6.55 ± 1.21	6.16 ± 0.92	0.4764	7.93 ± 1.18	7.61 ± 0.92	0.5841
	DHA (nmol/mg FW)	3.34 ± 0.85	0.96 ± 0.64	1.92E-06 ***	1.46 ± 0.63	1.07 ± 0.60	0.05983	0.76 ± 0.37	0.46 ± 0.27	0.2958
	ASC / DHA ratio	3.52 ± 1.38	26.85 ± 69.17	0.0003117 ***	5.90 ± 4.05	11.62 ± 19.31	0.2066	12.28 ± 8.06	32.93 ± 41.68	0.4117
	Total glutathione (pmol/mg FW)	1071.53 ± 232.70	683.48 ± 168.46	1.26E-06 ***	771.49 ± 166.18	563.82 ± 59.98	1.80E-05 ***	1052.99 ± 322.03	800.23 ± 211.32	0.005761 **
	GSH (pmol/mg FW)	951.45 ± 226.35	652.07 ± 172.27	7.83E-05 ***	711.41 ± 155.45	540.07 ± 66.92	0.00029 ***	903.60 ± 257.63	739.99 ± 183.74	0.0349
	GSSG (pmol/mg FW)	60.04 ± 33.81	20.24 ± 19.47	1.19E-04 ***	29.59 ± 19.34	14.87 ± 12.04	0.01513 *	74.70 ± 46.20	22.81 ± 15.41	1.20E-05 ***
	GSH / GSSG ratio	22.12 ± 14.37	66.11 ± 64.79	0.008856 **	57.62 ± 114.87	115.25 ± 179.23	0.05827	16.46 ± 10.63	47.33 ± 30.95	1.80E-05 ***
	NAD^{+} (pmol/mg FW)	42.68 ± 26.54	35.82 ± 14.78	0.5281	24.64 ± 13.62	20.01 ± 14.94	0.3378	39.12 ± 14.70	39.90 ± 17.90	1
	$NADP^{+}$ (pmol/mg FW)	15.70 ± 9.19	13.06 ± 6.26	0.3753	1.69 ± 1.83	10.64 ± 6.21	0.0001351 ***	3.04 ± 2.09	9.11 ± 4.75	0.0002695 ***
CROWN	Total ascorbate (nmol/mg FW)	4.74 ± 1.37	2.03 ± 0.36	9.01E-06 ***	2.53 ± 0.55	1.510556 ± 0.3797441	2.57E-05 ***	2.64 ± 0.37	1.50 ± 0.42	8.64E-07 ***
	ASC (nmol/mg FW)	5.15 ± 0.89	2.17 ± 0.37	4.41E-10 ***	2.72 ± 0.51	1.66 ± 0.40	1.15E-05 ***	2.44 ± 0.37	1.42 ± 0.38	1.64E-06 ***
	DHA (nmol/mg FW)	0.19 ± 0.09	0.10 ± 0.03	0.4	0.18 ± 0.14	0.04 ± 0.01	0.3333	0.23 ± 0.17	0.12 ± 0.07	0.08391
	ASC / DHA ratio	34.31 ± 21.92	20.78 ± 7.58	0.6286	23.15 ± 19.52	40.91 ± 26.54	0.6667	30.84 ± 54.86	19.12 ± 17.94	0.951
	Total glutathione (pmol/mg FW)	738.68 ± 157.79	603.48 ± 164.18	0.03202 *	642.88 ± 187.51	538.17 ± 107.46	0.1182	1221.19 ± 270.75	678.67 ± 213.08	4.60E-07 ***
	GSH (pmol/mg FW)	675.23 ± 163.52	547.14 ± 149.94	0.06826	619.97 ± 186.62	535.11 ± 106.30	0.2621	1017.54 ± 231.55	584.22 ± 195.21	2.53E-06 ***
	GSSG (pmol/mg FW)	33.15 ± 14.94	8.02 ± 5.71	4.49E-06 ***	9.07 ± 7.03	6.22 ± 3.82	0.3163	101.82 ± 50.98	47.23 ± 22.73	9.59E-05 ***
	GSH / GSSG ratio	26.38 ± 16.43	146.88 ± 199.42	3.72E-05 ***	392.34 ± 1121.34	134.50 ± 110.42	0.6623	13.98 ± 12.34	15.12 ± 8.30	0.2142
	NAD^{+} (pmol/mg FW)	109.22 ± 16.54	58.49 ± 19.48	2.95E-08 ***	62.77 ± 23.82	40.69 ± 14.05	0.005631 *	96.91 ± 19.84	56.05 ± 13.61	1.12E-07 ***
	$NADP^{+}$ (pmol/mg FW)	9.87 ± 3.70	2.024 ± 1.40	7.71E-08 ***	4.17 ± 2.18	2.65 ± 1.86	0.05945	7.44 ± 2.13	2.96 ± 2.04	1.77E-07 ***

** indicates significant differences at p < 0.05, ** at p < 0.01, and *** at p < 0.001.*

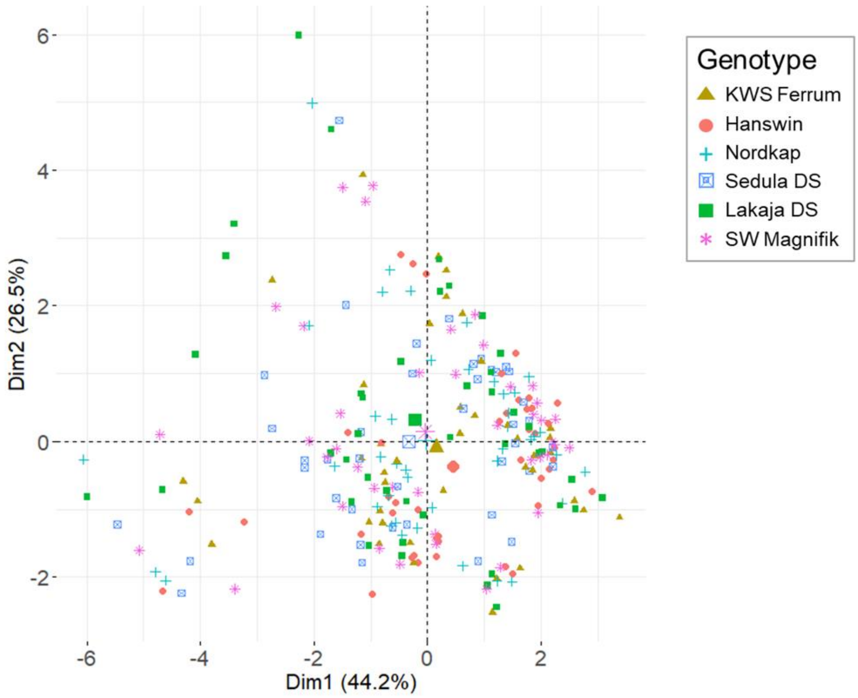

Figure S1. Principal component analysis (PCA) of antioxidants, measured throughout the experiment under constant low-temperature (CLT) and prolonged higher low-temperature (PHLT) treatments, grouped by genotype.

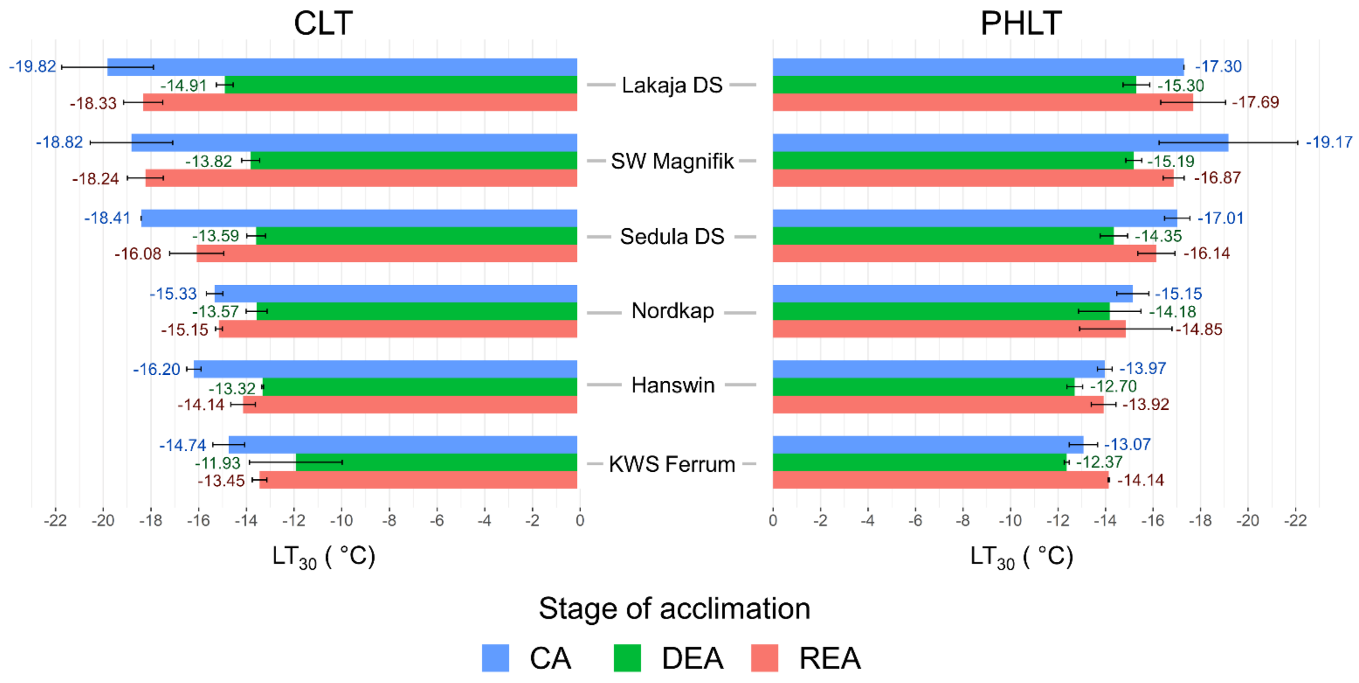

Figure S2. LT_{30} (temperature, under which 30% of plants die) values of six different winter wheat genotypes after cold acclimation (CA), deacclimation (DEA) and reacclimation (REA) under constant low-temperature (CLT) and prolonged higher low-temperature (PHLT) treatments. This data was discussed by Vaitkevičiūtė et al. (2022) [1].

References

1. Vaitkevičiūtė, G.; Aleliūnas, A.; Gibon, Y.; Armonienė, R. The Effect of Cold Acclimation, Deacclimation and Reacclimation on Metabolite Profiles and Freezing Tolerance in Winter Wheat. *Front. Plant Sci.* **2022**, *13*, doi:10.3389/fpls.2022.959118.
